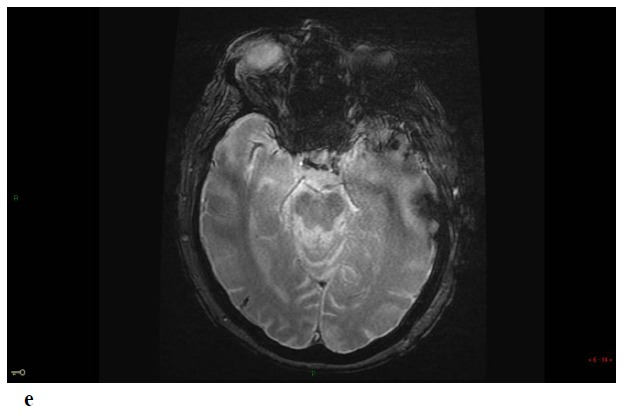# Convexity Subarachnoid Hemorrhage, Pseudomonas Aeruginosa (PA) Infective Endocarditis and Left Atrial Appendage Occluder (LAAO) Device Infection. A Case Report

**DOI:** 10.2174/1874440001711010026

**Published:** 2017-05-22

**Authors:** Monique Boukobza, Ibtissem Smaali, Xavier Duval, Jean-Pierre Laissy

**Affiliations:** 1 Department of Radiology, Assistance Publique-Hôpitaux de Paris, Bichat Hospital, Paris, France; 2 Department of Infectious Diseases, Assistance Publique-Hôpitaux de Paris, Bichat Hospital, INSERM Clinical Investigation Center 007, (S.T., X.D.) and INSERM U738, (C.L., X.D.) Université Paris Diderot, Sorbonne Paris Cité, France; 3 Department of Radiology, Assistance Publique-Hôpitaux de Paris, Paris, France; INSERM U1148, Paris, France; University Paris 7, Bichat Hospital, Paris, France

**Keywords:** Infective Endocarditis, Convexity subarachnoid hemorrhage, T2-GRE images, Mycotic aneurysm, Pseudomonas Aeruginosa, Left atrial appendage occluder

## Abstract

An 83 year-old-man with left atrial appendage occluder (LAAO) developed Pseudomonas Aeruginosa (PA) infective endocarditis. MRI at day 3 of onset showed distal small infarcts in both middle cerebral arteries and left postero-inferior cerebellar artery territories. MRI at day 6 revealed two sites of convexity subarachnoid hemorrhage (cSAH). MRA and CTA failed to reveal a Mycotic aneurysm.

The radiologic findings favor the assumption of necrosis of distal branches of mca or of pial arteries wall.

This case present three unusual features: the presence of localized cSAH after initiation of antibiotherapy without mycotic aneurysm being individualized; the late occurrence of infective endocarditis after LAAO implantation; the very rare occurrence of PA in prosthetic infections.

## INTRODUCTION

Subarachnoid hemorrhage (SAH) complicating a mycotic aneurysm (MA) has been reported in only 2- 5% of patients with infective endocarditis (IE) [1], while SAH occuring in the absence of an MA remains an infrequent feature of IE [2-5].

We report a case of an IE of the left atrial appendage occluder (LAAO), caused by Pseudomonas Aeruginosa (PA) and complicated by cerebral ischemia and 2 sites of convexity SAH (cSAH).

### CASE REPORT

An 83 year-old-male presented to the emergency room with fever and confusion. He had a stable ischemic heart disease, permanent atrial fibrillation (AF). After an unsuccessful radiofrequency ablation of AF three years earlier, he had an endovascular occlusion of the left atrial appendage (LAA) 30 months before and was left on antiplatelet therapy (lysine acetylsalicylate, 75mg/day).

At admission, confusion, neck stiffness, left sided hemiparesis and endophtalmia with purulent right eye secretions were noted. Body temperature was 40°C and arterial pressure was 153/93. A systolic aortic murmur without abolition of B2 and a systolic mitral murmur were present. An arbitrary antibiotherapy was started on (cefotaxim + ciprofloxacin + amikacin).

The initial work-up revealed in the peripheric blood a WBC count of 9200/mm3, an Hb of 10g/dl, a platelet count of 165 000G/l, a CRP at 182 mg/l, and an isocoagulant status (INR: 1.26, CTA: 1.19). PA was detected in blood cultures (time of positivity after inoculation of 10 and 15 hours).CSF showed a cell count of 40 cells/µl (90% of neutrophils), and rare colonies of PA.

Non-enhanced Brain CT at admission was normal. MRI at day 3 showed multiple small foci of ischemic lesions within the distal middle cerebral arteries (MCA) territories, the pons and the left postero-inferior cerebellar artery territory Fig. (**1**-**a**-**b**). There was no hemorrhagic lesion.

Transthoracic echography (TTE) and transesophageal echography (TEE) showed a huge mobile tissular lesion located on the LAAO, corresponding either to septic vegetation or to thrombi. The mitral leaflets were heavily calcified

A definite IE by PA – according to the modified Duke’s criteria - complicated with endophtalmia, cerebral emboli and meningitis was diagnosed and adequate antibiotherapy was started on.

Repeat MRI at day 6 showed besides the lesions described before, two sites of localized SAH on FLAIR and T2-GRE images at the left temporal convexity -extending to the external part of the sylvian fissure- and at a right temporal sulcus, and revealed a minor hemorrhagic transformation of the rolandic infarct Figs. (**1**-**c**-**d**-**e**) 3D-TOF MR Angiography of the Willis circle showed a dolicho-basilar trunk.

Brain CT-Angiogram (CTA) failed to reveal an MA or other cause of SAH, and showed a stenosis (50%) of both internal carotid arteries.

The 18-F-FDG PET scan was compatible with the diagnosis of IE of the LAAO with septic localizations at the right eye.

Per-operative TEE confirmed the diagnosis of a huge plurilobular formation fixed to the LLAO, a moderate to severe mitral regurgitation because of a P2-A2 mitral prolapse and images of small vegetations on P2. The LAAO and the native mitral valve apparatus were removed and a mitral biological prosthesis was implanted.

The patient was rapidly discharged from the operative room with stable hemodynamics. However, he died 3 days later from a refractory cardiogenic shock.

## DISCUSSION

The reported case presents three unusual features: firstly, the presence of localized cSAH without MA being individualized; secondly the late occurrence of IE after LAAO implantation; finally PA being an infrequent bacterium responsible for prosthetic infections.

SAH without associated MA has been rarely reported in IE.

Krapf [2] reported a case of IE with an abundant amount of blood in the sylvian fissure, and thirteen days later, the patient developed a huge parenchymal hematoma. At surgery, the proximal segment of the MCA was necrotic, and no MA was identified. A follow-up angiography did not reveal any vascular lesion. The patient survived with a left sided deficit.

Chukwudelunzua [3] identified 8 cases of SAH among 489 patients with IE (1%), diagnosed by Brain-CT. Cerebral angiography or autopsy did not reveal any MA in 6 cases. SAH was localized at the fronto-parietal sulci in 3, at the sylvian fissure in 2 and at the basal cisterns in1. In 1 /29 cases of spontaneous localized cSAH the SAH was related to IE, furthermore associated with cerebral microbleeds [6]. Recently, Graff-Radford [5] reported a prevalence of 10% of IE in a series of 88 patients presenting with non-traumatic cSAH.

SAH are usually attributed to ruptured MA, even when no aneurysm is demonstrable because they are sometimes obliterated by the hemorrhage that they produce, hence their angiographic and even pathologic demonstration is not always possible.

Our patient had no image confirming rupture of MA on CTA. The SAH was mild to hide any aneurysm and the patient was only under antiplatelet therapy, ruling out any suspicion of anticoagulation overdose responsible for the bleeding.

Septic emboli can be responsible for erosive arteritis causing rupture of the artery wall and intracerebral hemorrhage. This eventuality occurs during uncontrolled infection, particularly with virulent organisms such as PA, which can be the case of our patient who continued to have septic signs although antibiotherapy had been instituted.

Furthermore, in the current case, the concomitance of multiple distal infarcts and 2 sites of cSAH lead us to believe that an erosive arteritis secondary to the bacterial infection was responsible for wall rupture of distal arteries. Nevertheless, rupture of small pial artery as a cause of SAH had been previously demonstrated in an autopsy report [7]. Rupture of small pial arteries might also be responsible of cSAH in the setting of IE.

Furthermore, CAA is the most commonly reported cause of cSAH and in this eventuality, the most common location for cSAH is the fronto-parietal area [8]. In reversible vasoconstriction syndrome (RVCS), the second commonest cause as in cerebral venous thrombosis (CVT) and PRES, cSAH may be extensive along both convexities [9-11]. In the reported case, cSAH was present at both temporal convexities. Further studies are necessary to evaluate the occurrence and the imaging features of cSAH related to IE.

On the other hand, this patient was followed for ischemic heart disease and was under aspirin for long years. He had never presented hypersensitive clinical manifestations and no worsening of his coronary artery disease. That why laboratory tests were not indicated for him. Recent studies have shown that only short-term use of low-dose aspirine is associated with increased risk of SAH [12].

IE located at the LAAO is an extremely rare complication. Two recent studies about feasibility and safety with the fourth generation watchman LAAOs failed to detect device infection [13]. Only one case of infection associated with atrial appendage occluder, occurring a few days after implantation has been reported (*staphylococcus aureus*) and was related to lack of sterile conditions during the procedure [9]. IE due to gram-negative bacilli (GNB) represents 4% of all IE and PA accounts for only about 3% of IE due to GNB [14].

PA endocarditis is usually associated with the use of intravenous drugs and with prosthetic devices [15]. PA infects mainly elderly immunodepressive patients and is frequent in intensive care unit. We suspected that our patient contracted the PA either from a recent stay at hospital after blood transfusion, or *via* ocular contamination, as he had a severe endophtalmia.

To the best of our knowledge, this is the first reported case of localized SAH secondary to PA IE in a patient after LAAO system implantation.

## CONCLUSION

Localized cSAH without MA and in the absence of anticoagulation is a very rare neurological complication of IE.

This case of late prosthetic IE demonstrates that patients with LAAO are at risk to develop infection of their device and should benefit from the same preventive recommendations as the high –risk populations.

Finally, localized cSAH should suggest IE when occurring in patients with implanted heart devices.

## Figures and Tables

**Fig. (1):**
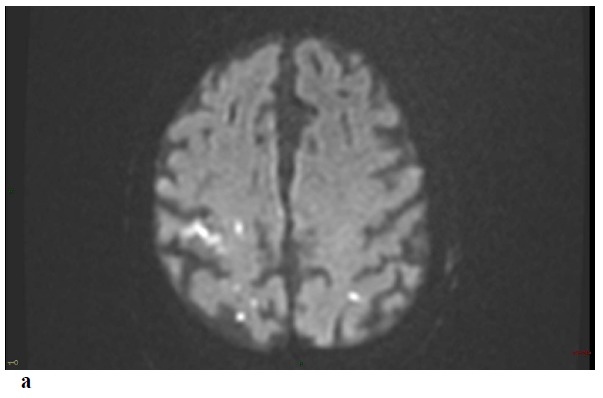
Axial DWI shows acute small embolic infarcts in territories of both middle cerebral arteries distal territory (**a**) and of the left postero-inferior cerebellar artery (**a**-**b**). Axial FLAIR, fluid attenuated inversion recovery (**c**) and axial T2 GRE images (**d**-**e**) show acute SAH filling partially the left sylvian fissure, the adjacent temporal convexity and a small sulcus at the right temporal convexity.

**Figure:**
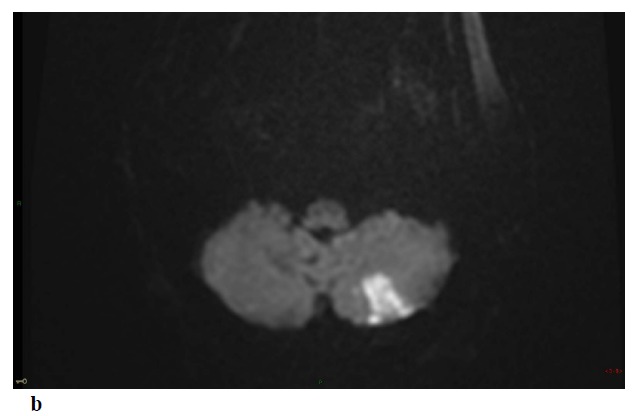


**Figure:**
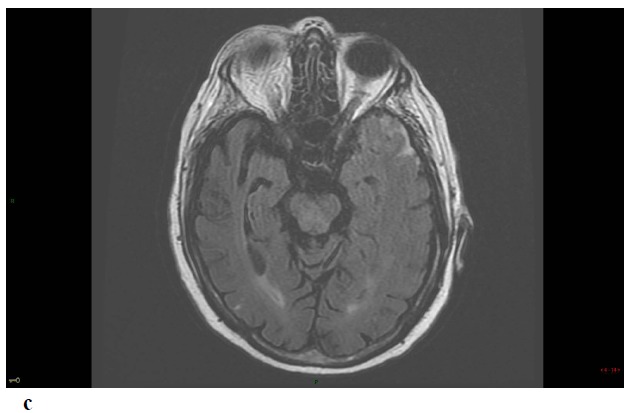


**Figure:**
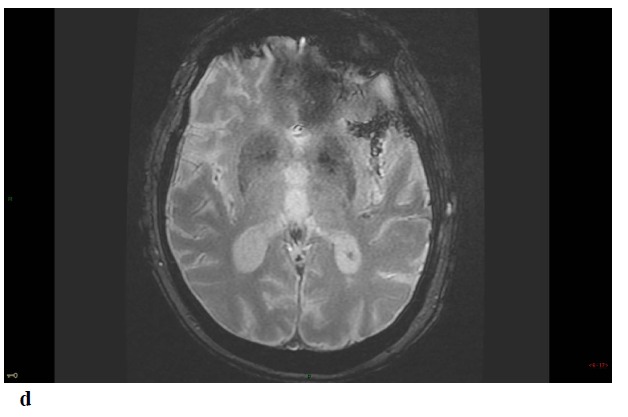


**Figure:**